# Mixed infection by *Cryptococcus neoformans* and *Cryptococcus gattii* and coinfection with paracoccidioidomycosis in PLHIV

**DOI:** 10.1016/j.mmcr.2022.01.006

**Published:** 2022-01-27

**Authors:** Marcela de Faria Ferreira, Fabio Brito-Santos, Pedro Henrique Nascimento Theodoro, Marcos de Abreu Almeida, Marcia dos Santos Lazera, Luciana Trilles

**Affiliations:** a National Institute of Infectious Disease Evandro Chagas, Oswaldo Cruz Foundation, Rio de Janeiro, 21040-900, Brazil; b Oswaldo Cruz Institute (IOC), Oswaldo Cruz Foundation, Rio de Janeiro, 21040-900, Brazil

**Keywords:** Cryptococcosis, HIV, Paracoccidioidomycosis, Cryptococcal antigen (CrAg), Lateral Flow Assay (LFA)

## Abstract

We present a rare condition of mixed *C. neoformans* and *C. gattii* infection in a person living with HIV with false-negative CrAg LFA in the CSF and co-infection with paracoccidioidomycosis. Signs and symptoms are relative to respiratory tract and skin, confounding with other opportunistic disease. After negatives CrAg LFA and Indian ink staining in CSF, there was isolation of *C. gattii* in sputum and *C. neoformans* in CSF, in addition to reagent serology (double immunodiffusion) for PCM with 1/16 titer. The patient was treated with amphotericin B and TMP-SMX with good clinical response and recovery of cellular immunity after initiation of antiretroviral therapy.

## 1 Introduction

A great diversity among *Cryptococcus* isolates has been described globally and each subtype has been associated with different patterns of virulence and geographic distribution [1]. Cryptococcosis by species of the *Cryptococcus neoformans* complex, most of the molecular type VNI, is a common fungal infection in people living with HIV (PLHIV), and the *C. gattii* species complex is common in immunocompetent individuals, mostly by molecular type VGII, in tropical climate regions, but rarely described in PLHIV in Southeastern Brazil [2]. The lateral flow assay (LFA) technique for detection of cryptococcal antigen (CrAg) has high sensitivity and specificity in serum and cerebrospinal fluid (CSF) for all serotypes of *Cryptococcus* spp [3]. Co-infections with other opportunistic diseases are common in PLHIV, however, weakly associated with paracoccidioidomycosis (PCM) [4]. We present here a rare case of PLHIV with mixed infection by *C. neoformans* VNI and *C. gattii* VGII, and PCM.

## 2 Case presentation

A 31-year-old male, born in Rio de Janeiro, Brazil, employee of an animal feed industry, attended at a referral center for infectious diseases in the same city, with seven months of cough, asthenia, progressive dyspnea, fever and skin lesions. No history of travel, exposure to decaying wood, or illicit drug use. He reported that two other employees were diagnosed with “pigeon disease”.

**Fig. 1:**
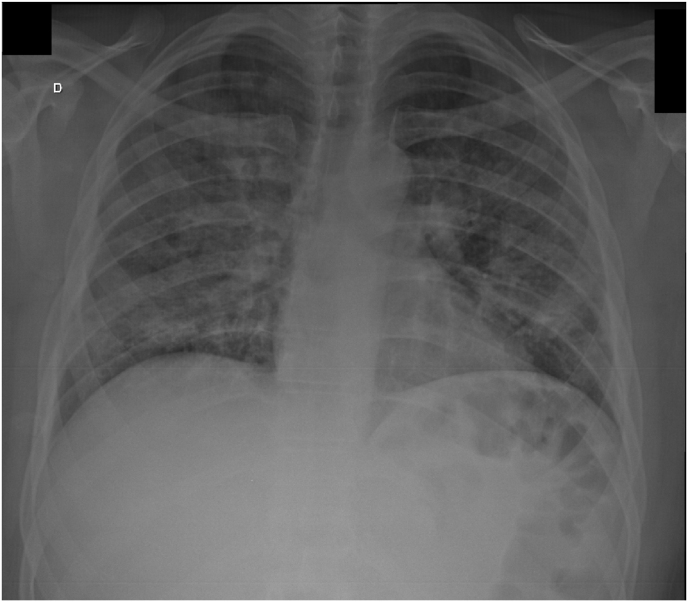
Chest X-ray with diffuse bilateral reticular infiltrate.

On day 0: physical examination revealed tachydyspnea, papules on the trunk, and oral candidiasis. There were no neurological signs or symptoms at admission. Relevant complementary tests showed a rapid HIV positive test, CD4 count 50 cells/mm³, hypoxemia on arterial blood gas (pO2 63), chest X-ray with diffuse bilateral reticular infiltrate (Fig. 1) and positive LFA CrAg in the serum.

He was hospitalized on day 0 and underwent blood culture, induced sputum, serology for PCM and histoplasmosis, and lumbar puncture (LP). The CSF had normal cytology and biochemical markers (50 mg/dl glucose, 49.1 protein, 2 cells, 100% mononuclear), negative CrAg LFA and India ink staining. Molecular test for *Mycobacterium tuberculosis* and BAAR (alcohol acid resistant bacillus) were negative in the induced sputum. Skin lesions have not been investigated. Treatment for pneumocystosis (PCP) with trimethoprim-sulfamethoxazole (TMP-SMX), 15 mg/kg/day of TMP, divided q6hs and preemptive treatment for isolated cryptococcal antigenemia with fluconazole 800 mg/day was initiated.

Day 4: Skin lesions improved on the fourth day, as did dyspnea, but the patient complained of headache. At that time, blood cultures for common bacteria were negative, double immunodiffusion for histoplasmosis was non-reactive, and double immunodiffusion (ID) for PCM was reactive with a titer of 1/16.

Day 11: The patient presented clinical improvement and was discharged from hospital with oral TMP – SMX 160/800 mg/day, due to its spectrum against PCM and because the medical team disregarded the diagnosis of PCP, and preemptive fluconazole 800 mg/day until complete two weeks, and then 400 mg/day por more eight weeks, according to Brazilian consensus.

During outpatient follow-up, blood cultures for mycobacteria and fungi were negative. After 5 weeks of incubation of CSF and induced sputum on Sabouraud 2% glucose and niger seed agar, *C. neoformans* was isolated from CSF and *C. gattii* isolated from induced sputum. The complexes were identified by canavanin-glycine-bromothymol (CGB) medium.

The patient was recalled to the hospital and submitted to another LP, with CrAg LFA persistently negative, even after dilution (up to 1/100) to exclude a prozone effect. A pre-heat CSF treatment was performed to increase the sensitivity of LFA, without changing the outcome. The isolates were confirmed by molecular analysis with MLST (multilocus sequencing type): VNI ST93 and VGII ST306.

He underwent induction treatment with amphotericin B deoxycholate 50 mg/day, followed by consolidation and maintenance treatment with fluconazole 400 mg/day. TMP-SMX was continued at 160/800mg/day until CD4 recovery.

Antiretroviral therapy with Tenofovir 300mg / Lamivudine 300 mg once daily and Dolutegravir 50 mg once daily was started six weeks after the start of the induction phase. After seven months of treatment, the patient recovered immunity with a CD4 cell count of 870 cells / mm³, an undetectable viral load and asymptomatic.

## 3 Discussion

The first atypical fact of this case is the isolation of both *C. neoformans* and *C. gattii* complexes in the same patient, in Rio de Janeiro, Brazil. This is the first report of mix infection with the two species complexes of cryptococcosis. The epidemiology of *C. neoformans* is well characterized, with VNI the most common worldwide and in Brazilian Southeast region, where has a high prevalence of *C. neoformans* associated with AIDS, but *C. gattii* is not common in this context [5]. *C. gattii* has historically been considered as a pathogenic agent of apparently immunocompetent patients, with VGII the most common within this species complex. In Brazil, VGII is common in North and Northeast, but not in Southeast and South region [2]. Diversity among *Cryptococcus* ST has been described all world, with correlation among virulence and geographical areas. VNI/ST93 is the most common in PVHIV and considered with high or intermediate virulence [5]. VGII/ST306, until moment, was identified once in São Paulo, a Brazilian Southeast region [6], reveling a dispersion of these agents and a potential to create a new endemic area of *C. gattii*.

The second atypical fact is the discrepancy between CrAg LFA negative in CSF with positive culture. Sensitivity for serum LFA among persons with cryptococcal meningitis was 99,6%, but the LFA technique in CSF with positive culture has high sensitivity, reaching 100% in published papers [3]. LFA false negative with positive culture has been reported only in cryopreserved CSF [7] and in a case of deficient capsule *Cryptococcus* spp [8]. In the present case, fresh CSF was used and was the first case of negative CrAg LFA with positive culture in our Institute. The long time the strains take to grow under laboratory conditions can be explained by the hypothesis that these strains are less virulent, as cryptococcal yeasts with genetic mutations grow slower [9]. Other hypothesis is the low fungal burden. More detailed genetic studies could clarify this fact.

The positive serology for PCM may correspond to an initial PCM infection or to an endogenous reactivation due to the low CD4 cells count. Cryptococcosis is frequently described along with pulmonary tuberculosis or even PCP in HIV patients [[10], [11]], but cryptococcosis/PCM co-infection is rarely diagnosed, with very few reported cases [[4], [12]]. PCM is usually diagnosed in patients from rural areas and mostly not related to immunosuppressive diseases. However, a recent highway construction triggered an ongoing PCM outbreak in the metropolitan area of Rio de Janeiro, Brazil, overlapping the geographical region of majority of the HIV cases [13]. The initial symptoms are nonspecific and could be due to cryptococcosis (*C.* gattii is known to cause outbreaks and the patient reported that two co-workers had “pigeon disease”) and PCM. PCM infection was early treated with SMX-TMP administration because of the primary diagnosis suspicion of PCP, what probably prevent the disease evolution and PCM specific symptoms.

In conclusion, cryptococcosis can mimic several other diseases, especially in pulmonary cases, with differential diagnosis of PCP and tuberculosis. In the context of AIDS, even without neurological symptoms, meningitis should be ruled out by lumbar puncture. The *C. neoformans* and *C. gattii* complexes can infect a single individual at the same time and *C. gattii* can cause disease in previously unrecognized endemic areas. Further investigations should be made to understand the false negative results of the LFA technique.

## Sources of funding for your research

There are none.

## Consent

Written informed consent was obtained from the patient or legal guardian(s) for publication of this case report and accompanying images. A copy of the written consent is available for review by the Editor-in-Chief of this journal on request.

## Declaration of competing interest

There are none.
